# Microneedle-Based Device for Biological Analysis

**DOI:** 10.3389/fbioe.2022.851134

**Published:** 2022-04-21

**Authors:** Huiting Lu, Shah Zada, Lingzhi Yang, Haifeng Dong

**Affiliations:** ^1^ Department of Chemistry, School of Chemistry and Biological Engineering, University of Science & Technology Beijing, Beijing, China; ^2^ Marshall Laboratory of Biomedical Engineering Research Center for Biosensor and Nanotheranostic, School of Biomedical Engineering, Health Science Center, Shenzhen University, Shenzhen, China

**Keywords:** microneedles, biological analysis, transdermal sampling, biomarker capture, detection, bio-signal recording

## Abstract

The collection and analysis of biological samples are an effective means of disease diagnosis and treatment. Blood sampling is a traditional approach in biological analysis. However, the blood sampling approach inevitably relies on invasive techniques and is usually performed by a professional. The microneedle (MN)-based devices have gained increasing attention due to their noninvasive manner compared to the traditional blood-based analysis method. In the present review, we introduce the materials for fabrication of MNs. We categorize MN-based devices based on four classes: MNs for transdermal sampling, biomarker capture, detecting or monitoring analytes, and bio-signal recording. Their design strategies and corresponding application are highlighted and discussed in detail. Finally, future perspectives of MN-based devices are discussed.

## Introduction

Blood sampling has become a common method in modern medical diagnosis due to its high efficiency and low cost. Many biometric tests are based on blood samples. However, the invasive sampling methods always accompanied subcutaneous acupuncture or finger pricks that usually lead to a decrease in patient compliance (Ayala et al., 2009). Meanwhile, blood sampling usually has to be performed by professionals. Although numerous efforts have been made in minimizing professional requirements during operation, the pain and discomfort still exist in the frequent finger-prick sampling with easy-to-use glucose meters. In addition, irregular operation and usage of unsterilized needles may pose a risk of blood-borne transmission of various biohazard pathogens (Sagoe-Moses et al., 2001). The disposal of sharp solid waste after blood sampling is also a problem.

Dermal interstitial fluid (ISF) is a portion of the fluid that seeps into the interstitial tissues from the arterial ends of capillaries. ISF acts as an intermediary between cancer cells and the circulatory system. Previous studies have proved that the composition and concentration of electrolytes, small molecules, and proteins in ISF are similar to those in plasma (Kool et al., 2007; Halvorsen et al., 2017; Niedzwiecki et al., 2018). Furthermore, some of the systemic and skin-derived metabolite biomarkers in ISF are unique (Muller A. C. et al., 2012; Niedzwiecki et al., 2018). Therefore, ISF is considered a promising alternative biofluid source with a lot of health-related information. The microneedle-based devices provide a noninvasive way to access this information.

The skin, the body’s largest organ, is a rich resource supply of ISF. However, the extraction of this fluid is restricted by a skin barrier. The stratum corneum, which is 15–20 μm thick, effectively prevents foreign substances from penetrating the skin (Dervisevic et al., 2020). Microneedles (MNs) are miniaturized needles with few hundreds of microns in length (Waghule et al., 2019). They can traverse the epidermis corneum and further insert into the viable epidermis while escaping from contacting any pain-sensing neurons or dermal blood vessels (Larraneta et al., 2016b). The first MN concept was proposed by Gerstel and Place in 1970 (Gerstel et al., 1976), while its blooming started until the 1990s after the development of micro- and nanofabrication techniques (Ventrelli et al., 2015). Since then, MNs have been fabricated using various materials with different physicochemical properties (Ali et al., 2019). The application of MNs initially focused on transdermal delivery of drugs (Kathuria et al., 2018) and vaccines (Creighton and Woodrow, 2019). Then, the study of MNs has been explored in many other areas including blood sampling (Xue et al., 2018), biosensing (Tasca et al., 2019), cosmetic (Yang J. et al., 2019), and cancer therapy (Moreira et al., 2019). MN-based devices have many fascinating properties, such as painless and minimal invasiveness, which show a great potential in point-of-care testing (POCT).

**FIGURE 1:**
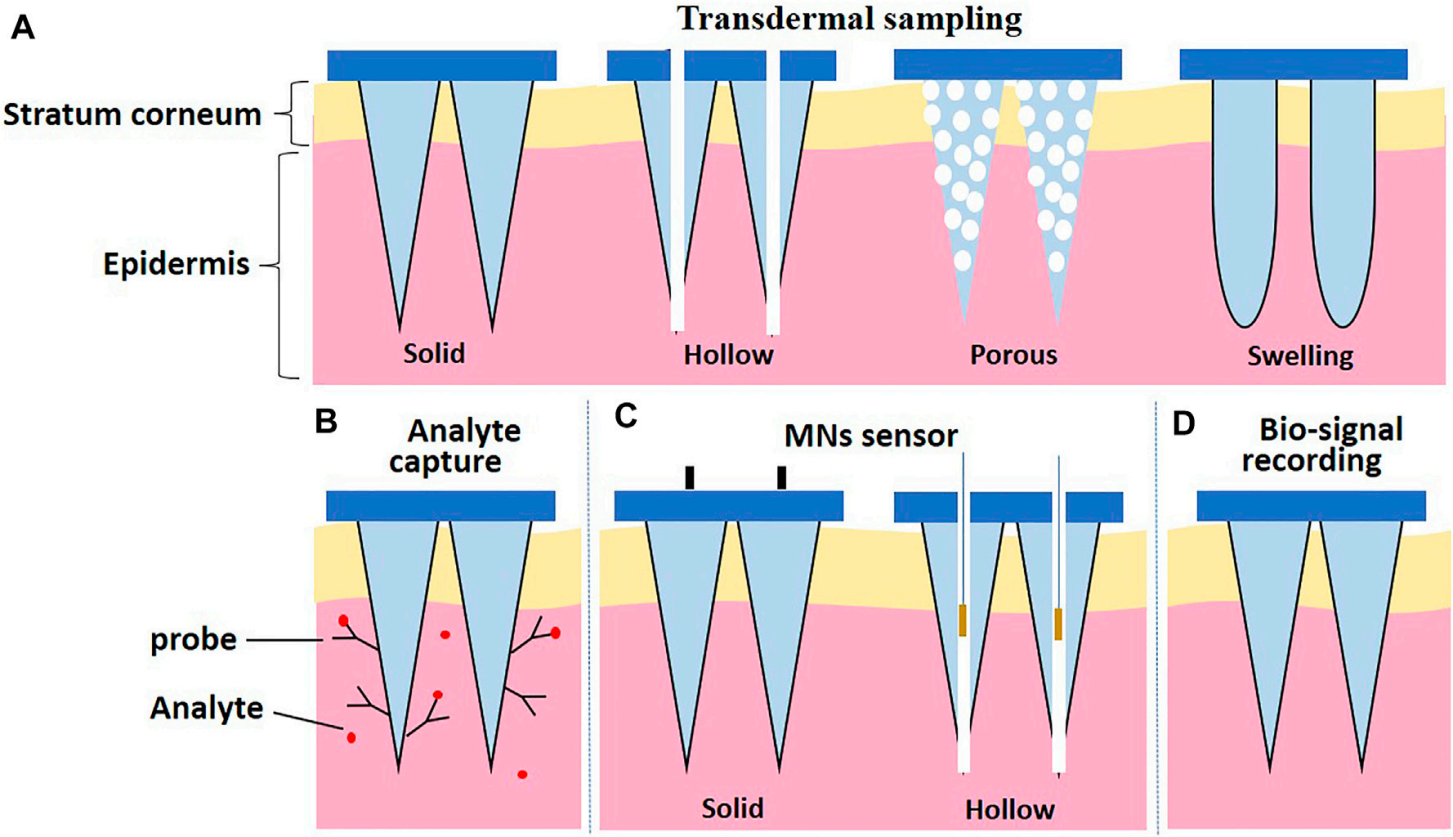
Schematic illustration of categories according to the design strategies of MNs. **(A)** Transdermal sampling. **(B)** Biomarker capture. **(C)** MNs sensor. **(D)** Bio-signal recording.

In general, MN-based devices can be classified into four categories according to their functional purposes (Figure 1): 1) MNs are used for transdermal sampling only with the corresponding materials analyzed by external methods 2) the MN array is designed to capture biomarkers selectively; 3) MNs are integrated in a sensor to detect or monitor the analytes within the skin; and 4) MNs are used for bio-signal recording platforms.

In the present review, we focus on the recent advances of microneedle-based devices. The four categories of MN-based devices and their applications are highlighted and discussed in detail. Finally, the future perspectives of MN-based devices are summarized.

## Microneedles for Transdermal Sampling

Four types of MNs (solid MNs, hollow MNs, porous MNs, and swelling MNs) have been used for transdermal sampling (Figure 1A). The properties of MNs for transdermal sampling are summarized in Table 1.

**TABLE 1:** Properties of MNs for transdermal sampling.

MN type	MN material	Manufacturing method	Sampling mechanism	Sampling amount/efficiency	Reference
Solid	Glass	Glass pulling	Pressure-driven convection	1–10 µl ISF	Wang et al. (2005)
	Polycarbonate	—	Capillary action	—	Sato et al. (2011)
	Stainless steel	Laser cutting	Capillary action	>2 µl of ISF in 1 min	Kolluru et al. (2019)
	Stainless steel	Micromachining	Capillary action	2.9 μl of ISF in 30 s	Kim et al. (2021)
Hollow	Stainless steel	Laser cutting	Inherent pumping mechanism	Up to 20 and 60 μl from humans and rats, respectively	Miller et al. (2018)
	Silicon	Micromachining	Capillary action	—	Mukerjee et al. (2004)
	Glycidyl methacrylate, trimethylolpropane trimethacrylate, and triethylene glycol dimethacrylate	Micromolding	Capillary action	Extracted simulated ISF within 5 s	Nicholas et al. (2018)
Porous	Cellulose acetate	Phase inversion	Capillary action	1.33 mg ISF in 10 min	Liu et al. (2020)
	PSF, PDA, and PEG	Phase inversion	Capillary action	1.41 mg ISF in 10 min	Liu P. et al. (2021)
	PDMS	Mold casting and salt leaching	Pressure-driven convection	0.019 µl/min (80 min after insertion: 0.080 µl/min)	Takeuchi et al. (2022)
Swelling	Acrylate-based hydrogels	Micromolding	Diffusion	6 µl of ISF in 10 min	Laszlo et al. (2021)
	MeHA	Micromolding	Diffusion	1.4 mg ISF in 1 min	Chang et al. (2017)
	MeHA	Micromolding	Diffusion	3.82 µl of ISF in 3 min	Zheng et al. (2020)
	GelMA	Micromolding	Diffusion	1.9 mg after 5 min	Zhu et al. (2020)
	PVA/CS	Micromolding	Diffusion	—	He R. et al. (2020)

PDMS, dimethylpolysiloxane; PSF, polysulfone; PDA, polydopamine; PEG, poly(ethylene glycol); MEMS, microelectro-mechanical system; MeHA, methacrylated hyaluronic acid; GelMA, gelatin methacryloy; PVA, polyvinyl alcohol; CS, chitosan.

**TABLE 2:** Properties of MNs for biomarker capture.

MN material	Manufacturing method	Target biomarker	Recognition mechanism	Sampling amount/efficiency	Reference
Gold-coated silicon	Deep reactive ion etching	AF-igg	Immunoaffinity	—	Corrie et al. (2010)
Gold-coated silicon	Deep reactive ion etching	Dengue Virus NS1 Protein	Immunoaffinity	8 µg/ml	Muller D. A. et al. (2012)
Polystyrene	Micromolding	Il-6 matricellular protein periostin	Immunoaffinity	0.33 pg/ml	Wang Z. et al. (2021)
			Immunoaffinity	—	
Al	Micromachined, electrochemical anodization	E2	Immunoaffinity	0.5–1,000 ng/ml (<1 min)	Kang et al. (2020)
PEG and PEGDA	Micromolding	TNF-α, IL-1β, IL-6	Immunoaffinity	—	Zhang et al. (2019)
Glass and ETPTA	Micromolding	LPS	Immunoaffinity	0.0064 EU/ml	Yi et al. (2021)
Poly(lactide)	Micromolding	miR-210	Watson−Crick base pairing	∼6.5 μl in 2 min	Al Sulaiman et al. (2019)
AuNWs, PMVE, and MA	Micromolding	EBV Cf DNA	Double spatial-orientated recognition	93.6%	Yang B. et al. (2019)
AuNWs, PMVE, and MA	Micromolding	EBV Cf DNA	Double spatial-orientated recognition and reverse iontophoresis	95.4%	Yang et al. (2020)

ETPTA, ethoxylated trimethylolpropane triacrylate; LPS, lipopolysaccharide; EBV Cf DNA, Epstein–Barr virus cell-free DNA; AuNWs, Au Nanowires; PMVE, polymethyl vinyl ether-alt; MA, maleic acid.

Solid MNs and hollow MNs, made by silicon, glass, and stainless steel, are usually used for transdermal sampling (Tariq et al., 2021). Normally, these types of MNs were punctured on the skin with the ISF collected by a vacuum pump or a well-absorbent film sucking to withdraw the tissue fluid for subsequent analysis (Wang et al., 2005; Nalbant and Boyaci, 2019). For example, Kolluru et al. (2019) designed a two-component system composed of a strip of paper on the back of stainless steel MN arrays. First, the MN arrays break the skin barrier and create micropores that allow the ISF to flow to the skin surface, in which the ISF can ultimately be sucked and reserved in the paper strips due to the capillary force. The optimized paper strip–stainless steel MN array is capable of >2 µl of ISF collection within 1 min safely and conveniently on a rat skin *in vivo*. Nicholas et al. (2018) integrated an enzyme-based colorimetric glucose sensor to a single hollow MN device and achieved rapid determination of glucose concentration in simulated ISF with physiological relevancy. The sharp tip of hollow MNs could help the MNs to better penetrate the skin. Through the capillary effect inside the skin, the uptake of ISF within 5 s is demonstrated.

Solid MNs and hollow MNs have greatly facilitated transdermal sampling. Nonetheless, instrumental assistance and multi-step operations are usually required to maintain a negative pressure during the preparation of MNs, which limits their application. Additionally, glass- or silicon-based MNs often suffer from brittleness, which poses a potential safety risk to patients and causes environmental hazards (Sivamani et al., 2007; Larraneta et al., 2016a). One solution to the aforementioned issues is to replace glass/silicon with porous polymers in MN fabrication. Porous polymer–based MNs exhibit various distinctive advantages including desirable biocompatibility, ease of processing, large cavity, and three-dimensional connected porous structure. Liu et al. (2020) developed a phase inversion route to prepare various polymer-based MNs using cellulose acetate (CA), polysulfone (PSF), polyethersulfone (PES), polylactic acid (PLA), etc. with high porosity and interconnected pore structures. The resultant porous polymer–based MNs can extract ISF rapidly. Liu P. et al. (2021) have demonstrated a facile and effective method for interconnected structured and hydrophilic porous polymer MN modification. The coating layer of PDA provides new sites for polymers post-functionalization, which is beneficial for further PEG modification on the polymer matrix. The imported PEG improved the porous structure hydrophilicity and enhanced the molecular resistance effect. In addition, this method could be promoted to other polymer materials such as PLA and PVDF. The strategy provides a new idea for the fabrication of hydrophilic and anti-adhesion porous polymer MNs. Takeuchi et al. (2022) developed a fluidic system that directly interferes with the porous MNs, in which a capillary pump was utilized inside the microfluidic chip for continuous ISF sampling. In detail, the developed device connects a standard microelectromechanical system (MEMS)-fabricated microfluidic chip with porous flexible MNs of PDMS. It achieved continuous flow control of phosphate-buffered saline (PBS). The development of the device is beneficial for long-term health monitoring applications based on minimally invasive and continuous bio-sampling.

**FIGURE 2:**
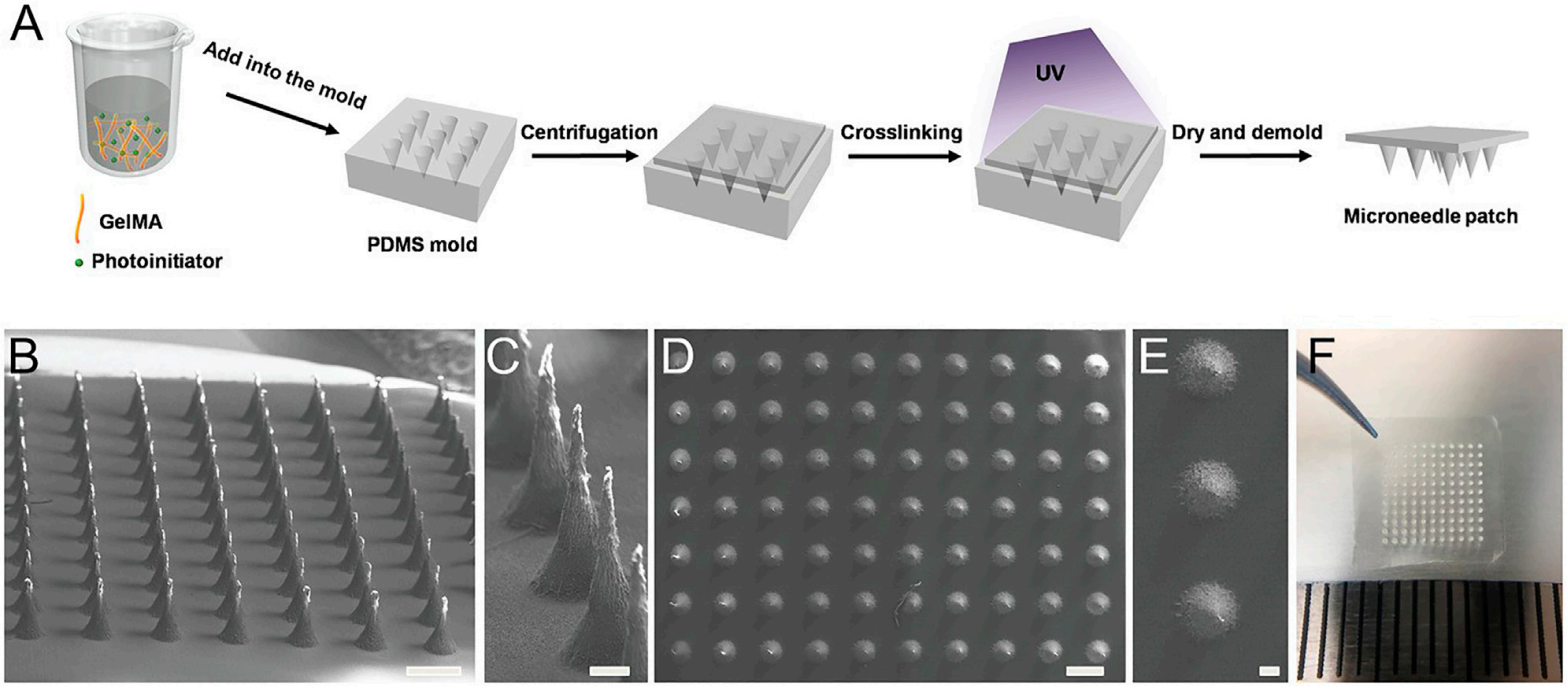
Preparation of the GelMA MN patch. **(A)** Schematic of the MN preparation process. GelMA aqueous solution (aq) was poured into a PDMS mold. After centrifugation and UV cross-linking, the patch was dried and demolded. **(B,C)** SEM images show the side view of the GelMA MN array and **(D,E)** SEM images from the top view. Aligned conical needles were formed with an approximate height of 600 µm and a bottom diameter of 300 µm. Scale bar: 500 µm in **(B)** and **(D)** and 100 µm in **(C)** and **(E)**. **(F)** Picture of the GelMA MN patch containing an 11 × 11 array of MNs over a 1 cm × 1 cm area. Reproduced with permission (Zhu et al., 2020). Copyright 2020, Wiley.

Another emerging good candidate for transdermal sampling is swelling MNs, which generally consist of cross-linked polymer networks and enable one-step ISF extraction without the aid of extra devices. Hydrogels are biocompatible and biodegradable. They are able to cause minimal tissue damage and little environmental threat after disposal (Herrmann et al., 2021). Chang et al. (2017) developed a methacrylated hyaluronic acid (MeHA)-based swellable MN platform, which can extract ISF efficiently for rapidly metabolic analysis. The MeHA patch could penetrate into the skin by a thumb press, extracting ≈1.4 mg ISF within 1 min, while maintaining structure integrity without leaving residues in the skin. A gelatin methacryloyl (GelMA)-based MN patch was developed in an 11 × 11 array fashion by a micromolding approach for minimally invasive ISF sampling (Figure 2) (Zhu et al., 2020). The properties of the patch could be adjusted by varying the GelMA prepolymer concentration and the crosslinking time. The swelling ratios of resulting patches are between 293 and 423%, and compressive moduli are between 3.34 and 7.23 MPa. The developed GelMA MN patch exhibits an efficient extraction of ISF. Laszlo et al. (2021) proposed two hydrogel-based MN arrays for dermal ISF proteomics sampling. The developed MN patches could restore their initial shape even after multiple mandatory washing steps to erase un-crosslinked polymers. Such a property could prevent signal-inhibiting effects and chromatographic interferences. The *in vitro* and *in vivo* biocompatibility was demonstrated. The collection of dermal ISF *in vivo* is also studied for subsequent proteomic applications. Zheng et al. (2020) developed a new osmolyte-composited swellable hydrogel MN for skin ISF extraction. The developed MN patch is composed of osmolytes such as maltose and hydrogels such as methacrylated hyaluronic acid. During extraction, the maltose would dissolve in the matrix and provide an osmotic pressure to enhance the diffusion rate of ISF to the hydrogel matrix. The microneedle prepared with the optimized formula could extract 7.90 µl ISF from pig ear skin and 3.82 µl ISF from mouse back skin within 3 min *in vitro*.

## Microneedles for Biomarker Capture

The MN array designed for biomarker capture is an emerging approach in modern medical diagnosis. As shown in Figure 1C, MN systems for biomarker capture usually contain identification elements or probes for the selection and capture of analytes.

**FIGURE 3:**
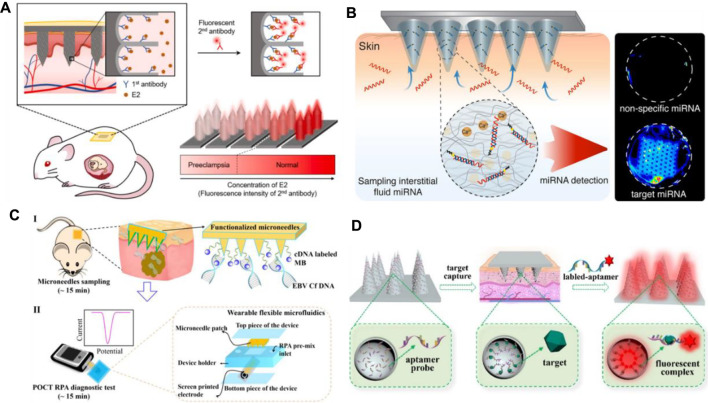
**(A)** Schematic diagram of immuno-functionalized MN patch for PE diagnosis by detecting E2 biomarker at the sub-nanogram level. Reproduced with permission (Kang et al., 2020). Copyright 2020, Elsevier. **(B)** Schematic representation of the hydrogel-coated microneedle platform during sampling of the interstitial fluid. Reproduced with permission (Al Sulaiman et al., 2019). Copyright 2019, American Chemical Society. **(C)** Schematic workflow of the assay, with MN patches sampling (I) and a POCT electrochemical microfluidic platform (II). Reproduced with permission (Yang B. et al., 2019). Copyright 2019, American Chemical Society. **(D)** Schematics of an aptamer-decorated porous MN array. The biomarkers could be captured by probes on the MNs. Reproduced with permission (Yi et al., 2021). Copyright 2021, Elsevier.

As shown in Table 2, the recognition mechanism of MNs for biomarker capture is mainly immunoaffinity. The recognition element is usually an antibody, oligonucleotide, or other proteins that can bind to a specific molecular motif. Thus, the target analyte can be effectively separated from the complex biological matrix. Figure 3A shows a typical example about this mechanism. The nanoporous MNs were functionalized with an antibody to selectively capture estrogen (E2) (a preeclampsia biomarker), following insertion into the skin, thereby enabling PE diagnosis by measuring biomarker concentrations based on the immunoassay method (Kang et al., 2020). To provide ultra-dense binding sites for the extraction and detection of trace amounts of the target analyte in the subcutaneous ISFs, a uniform nanopore structure was created on the tissue-penetrating aluminum (Al) MN array by controlled anodic oxidation. The immuno-functionalized nanoporous MN patch’s rapid, sub-nanogram-level sensing ability was verified by *in vitro* E2 detection tests and *in vivo* skin tests of experimental animals with response signals obtained within 1 min.

**TABLE 3:** Properties of MN-based sensor.

Analysis approach	Analysis object	Detection/continuous monitoring	Test subject	References
Electrochemical	Glucose	Detection	ISF samples	Strambini et al. (2015)
	Glucose	Continuous monitoring	Mice	Liu Y. et al. (2021)
	Glucose	Continuous monitoring	Artificial ISF	Bollella et al. (2019c)
	Glucose	Continuous monitoring	Phosphate-buffered saline	Invernale et al. (2014)
	Glucose	Continuous monitoring	Buffered saline	Ribet et al. (2018)
	Glucose	Continuous monitoring	Volunteers	Sharma et al. (2016)
	Glucose	Continuous monitoring	Volunteers	Kim et al. (2019)
	Glucose	Continuous monitoring	Healthy volunteers and T1D	Sharma et al. (2018)
	Glucose	Continuous monitoring	Rabbit	Lee et al. (2016)
	Glucose	Continuous monitoring	Mice	Chen et al. (2015)
	Lactate	Detection	Sensor-enclosing device	Wang Y. et al. (2020)
	Lactate	Detection	Mice	Li Q. et al. (2021)
	Lactate	Continuous monitoring	Artificial ISF	Bollella et al. (2019a)
	H_2_O_2_	Real-time monitoring	Living HeLa cells	Zhou et al. (2017)
	H_2_O_2_	Real-time monitoring	Mice	Jin et al. (2019)
	Potassium	Detection	Chicken and porcine skin	Parrilla et al. (2019)
	Urea	Detection	Artificial ISF and alginate epidermal/skin mimic	Senel et al. (2019)
	Alcohol	Real-time monitoring	Artificial ISF and mice skin	Mohan et al. (2017)
	Organophosphate	Detection	Mice skin	Mishra et al. (2017)
	Phenoxymethylpenicillin	Real-time monitoring	Volunteers	Rawson et al. (2019)
	Beta-lactam antibiotics	Continuous monitoring	Volunteers	Gowers et al. (2019)
	Levodopa	Continuous monitoring	Artificial ISF and mice skin-	Goud et al. (2019)
	Glucose, lactate	Detection	Solution	Calio et al. (2016)
	Glucose, lactate	Detection	Porcine kidney	Samper et al. (2019)
	Glucose, lactate	Detection	Artificial ISF	Bollella et al. (2019b)
	Myoglobin, troponin	Detection	Solution	Miller et al. (2016)
	HB, glucose, and lactate	Detection	Artificial ISF	Teymourian et al. (2020)
	Glucose, uric acid, and cholesterol	Detection	Solution	Gao et al. (2019)
SERS	Glucose	Detection	Skin phantom	Yuen and Liu (2014)
	Glucose	Detection	Mouse	Ju et al. (2020)
	MB	Detection	Skin phantom	Linh et al. (2021)
	pH	Detection	Agar gel skin phantom and human skin	Park et al. (2019)
Colorimetric	Glucose	Detection	Simulated ISF	Nicholas et al. (2018)
	Glucose	Detection	Mouse	Zeng et al. (2020)
	Glucose	Detection	Mice	Wang Z. et al. (2020)
	pH, glucose, uric acid, and temperature	Detection/monitoring	Rabbit	He et al. (2021)

T1D, participants with type 1 diabetes; HB, β-hydroxybutyrate; NO, nitric oxide; MB, methylene blue.

Apart from immunoaffinity, Watson–Crick base pairing has also been applied in MN systems for biomarker capture. Al Sulaiman developed alginate–peptide nucleic acid-coated MNs that are capable of sequence-specific capture of target miRNAs by Watson–Crick base pairing (Figure 3B) (Al Sulaiman et al., 2019). Attaching PNA oligomers to the hydrogel’s fibers enables specific sampling, purification, and release of the only nucleic acid fragments that are complementary to the PNA sequence. By simply adapting the PNA sequence, this system can be applied in any miRNA of interest.

Yang B. et al. (2019) provide another way of biomarker capture by MNs. They employed hydrogel MN patch for fast and easy *in situ* capture of Epstein–Barr virus cell-free DNA (EBV Cf DNA) from ISF in 15 min with a capture efficiency as high as 93.6% (Figure 3C). Detection of EBV Cf DNA was achieved by electrochemical recombinase polymerase amplification implemented wearable flexible microfluidics, with a detection limit of 3.7 × 10^2^ copies/μl. Animal experiments support the effectiveness of MNs for EBV Cf DNA capture. Later, they combined reverse iontophoresis and MN techniques and engineered a wearable epidermal detection system for rapid sensing of EBV Cf DNA (Yang et al., 2020). On account of the additive extraction effect of reverse iontophoresis and MNs, the engineered platform successfully separated cell-free DNA targets from ISF within 10 min, with a maximum capture efficiency of 95.4% and a threshold of 5 copies per µl. Captured cell-free DNA is directly used in the developed electrochemical microfluidic biosensor with a detection limit of 1.1 copies/µl.

Until now, MN arrays for capturing biomarkers have been reported by many groups. Corrie et al. (2010) first designed a MN-based device that captures circulating biomarkers in skin fluids in a painless manner with high specificity. They grafted a hetero-bifunctional PEG to the gold-coated microprojections, which reduced the unspecific protein binding and provided a spot for capturing attachment proteins. High specificity and high sensitivity antibody capture, extraction, and analysis have been achieved. Using the similar method, their groups realized the capture of dengue virus NS1 protein (Muller D. A. et al., 2012), recombinant *P. falciparum* rPf HRP2, and total IgG from the complexed matrix (Lee et al., 2014). Wang Z. et al. (2021) reported that functional biomolecules (such as antibodies) modified MNs that could penetrate the stratum corneum and periosteum, enabling the selective capture of protein biomarkers in the local ISF in a concentration-dependent manner. Due to the hydrophobic interaction between polystyrene and nonpolar residues on the protein, this interaction effectively traps the hydrophobic structures in antibodies and proteins and binds them to the polystyrene surface. Zhang et al. (2019) developed photonic crystal (PhC) barcodes integrated in encoded MNs for detecting ISF biomarkers. PhC barcode–loaded flexible MNs were elegantly fabricated by replication of dynamic ferrofluid casting micropatterns. When the prepared MNs are inserted into the skin, they could enrich certain biomarkers into their PhC barcode–modified probes. Therefore, sandwich immunocomplexes can be formed after the addition of corresponding fluorescent tags. The relative level of the biomarkers could be read out by the fluorescence signals of the barcode; at the same time, the types of biomarkers could be clearly discriminated by the reflection peaks of the PhC barcode.

It is worth noticing that aptamers can be potentially applied in MN arrays for biomarker capture. Aptamers are recognized as single-stranded oligonucleotides in 25–80 base length that exhibit specific binding affinity toward targets including amino acids, drugs, and protein biomolecules. In comparison with protein antibodies, aptamers have several advantages such as small size, strong binding affinity, high specificity, desirable biocompatibility, good stability, and lesser immunogenicity, making them widely used in the field of biomedicine (He F. et al., 2020; Ni et al., 2021). Aptamer-modified porous MN arrays are proposed to enable *in situ* enrichment and detection of biomarkers in ISF by Yi et al. (2021) (Figure 3D). Porous MN arrays were made by replicating negative molds containing SiO_2_ microspheres and UV-curable ethoxylated trimethylolpropane triacrylate (ETPTA). Since the MN array combines advantages of aptamers and porous structures, its surface area is remarkably increased to 6.694 m^2^/g, so a large amount of aptamer probes (0.9459 μM) can be fixed. Furthermore, due to capillary forces, the MN array can extract ISF into its porous structure. Subsequently, biomarkers are captured and detected without sample post-processing.

## Microneedle Sensor for Detecting or Monitoring Analytes

MN-based sensors have been employed widely for detecting or monitoring a diverse range of analytes as listed in Table 3.

### Microneedle-Based Electrochemical Sensor

As displayed in Table 3, the detection mechanism of a majority of MN-based sensors relies on electrochemical sensing technology.

**FIGURE 4:**
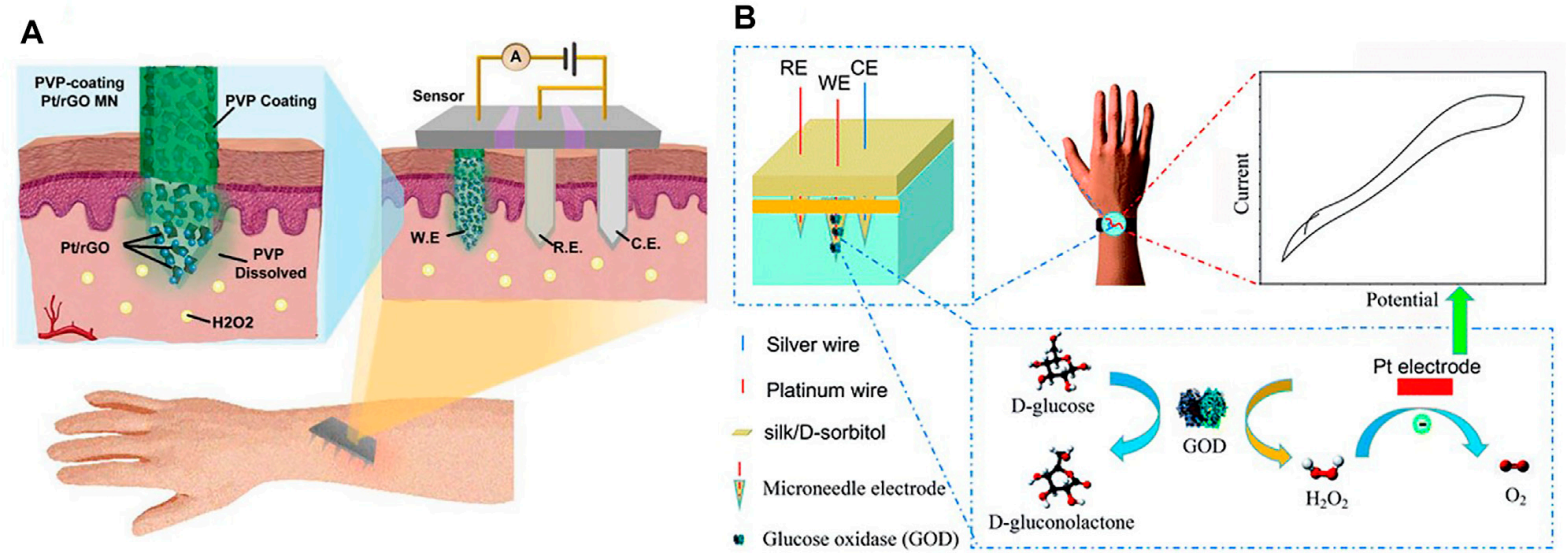
**(A)** Schematics for illustration of the transdermal H_2_O_2_ electrochemical biosensor on the basis of the conductive MN patch with Pt/rGO surface. Reproduced with permission (Jin et al., 2019). Copyright 2019, Wiley. **(B)** Schematics for the working mechanism of silk/D-sorbitol microneedle electrodes. Reproduced with permission (Zhao et al., 2020). Copyright 2019, Royal Society of Chemistry.

Solid MNs are commonly used in MN-based electrochemical sensors. First, the materials used for MN fabrications are highly conductive and thus can be directly used for analyte monitoring to construct enzyme-free electrochemical biosensors. Li et al. (2020) developed a MN-based electromagnetic generator with a magnetized MN-array for monitoring human motion. The magnetized MNs in triboelectric–electromagnetic hybrid generators were employed as the frictional layer of triboelectric generator (TEG) and served as the bendable magnetic poles of electromagnetic generator (EMG). Gold (Calio et al., 2016; Senel et al., 2019; Zhang et al., 2020; Zhao et al., 2020) and platinum (Zhou et al., 2017; Chinnadayyala et al., 2018a; Zhao et al., 2020) were usually used to fabricate MNs due to their good conductivity. An attractive glucose sensor using a nafion and platinum black-coated MN electrode array in three- and two-electrode configuration was reported (Chinnadayyala et al., 2018a). An enzyme-free electrochemical sensing platform based on a Pt-MN electrode functionalized with Au nanoparticle (Au-NP)-decorated polydopamine nanospheres (PDA-NSs) was explored in *in vitro* and *in vivo* detection of lactate in different biological samples (Li Q. et al., 2021). Goud et al. (2019) developed a MN sensing platform that relied on parallel simultaneous independent enzymatic-amperometric and nonenzymatic voltammetric detection of L-DOPA using different microneedles on the same sensor array patch. As a significant alternative of noble metal materials, carbonic materials or compositions were widely used for construction of MNs. Skaria et al. (2019) used micromolding techniques to construct solid MN sensors using poly(lactic acid)/carboxyl-multiwalled carbon nanotube (PLA/f-MWCNT) composites. Jin et al. (2019) developed a conductive MN patch for transdermal H_2_O_2_ electrochemical biosensing. The MN surfaces was modified with hybrid materials of Pt nanoparticles and reduced with graphene oxide (rGO) nanostructures that serve as an active H_2_O_2_ sensing modulus (Figure 4A). The detection sensitivity of MN electrodes was significantly improved by Pt/rGO, and MN was used as a painless transdermal tool for *in vivo* access.

Second, the solid MNs often functionalized with enzymes to convert analytes into detectable electrochemical signals. Oxidase (GOx) (Chen et al., 2015; Wang et al., 2021a) was commonly used to monitor glucose by converting glucose into gluconic acid and hydrogen peroxide H_2_O_2_. A flavin adenine dinucleotide glucose dehydrogenase/6-(ferrocenyl)hexanethiol/highly porous gold (FAD-GDH/FcSH/h-PG) MN-based biosensor was fabricated for minimally invasive sensing of glucose in artificial interstitial fluid (Bollella et al., 2019c). In addition, lactate oxidase (LOX) enzyme was also explored to develop MN-based electrochemical sensors. Bollella et al. (2019a) developed a mediated pain-free MN-based biosensor for the continuous monitoring of lactate in the ISF (Bollella et al., 2019a). Functionalization of the Au-MWCNTs/poly methylene blue (MB) platform with LOX enzyme by drop casting procedure enabled the continuous monitoring of lactate in artificial interstitial fluid and in human serum. Other enzyme MN electrochemical biosensors were also reported. Gold MNs (AuMNs) functioned with an epoxy- and ferrocene-functional polymeric mediator and covalently immobilized urease was used for urea sensing (Senel et al., 2019). Mohan et al. (2017) developed a skin-worn MN sensing device for the minimally invasive electrochemical monitoring of subcutaneous alcohol.

Hollow MNs could also be used for electrochemical sensing in which the transducers are integrated into the back of the hollow MN array to detect the substance (Jina et al., 2014). Strambini et al. (2015) prepared a self-powered painless MN biosensor for a highly accurate analysis of glycemia in ISF. The enzymatic glucose biosensor was coupled to the backside of the needle patch for a self-powered glucose analysis (*in vitro*) in the ISF with high accuracy, high reproducibility, and excellent linearity (0–630 mg/dl) in real time. Hollow MN-based sensors were also prepared by traditional electrochemical sensing element embedment in the hollow MNs. Compared to aforementioned MNs, this approach provides additional protection for the sensing elements. For example, MN-based organophosphorus hydrolase (OPH) sensors were reported for minimal-invasive measurement of transdermal threat compounds, organophosphate (OP) (Mishra et al., 2017). The sensor relied on the efficient biocatalysis of OPH on a MN-modified carbon paste array electrode and used fast square wave voltammetry (SWV) of the p-nitrophenol product of the enzymatic catalytic reaction of OPH to measure the OP with good selectivity. Teymourian et al. (2020) presented a MN platform for real-time ketone body monitoring based on a β-hydroxybutyrate (HB) dehydrogenase enzymatic reaction. This amperometric sensor for dual analytes is realized by filling the hollow microneedle tip with an appropriate electrode material, where simultaneous HB/glucose detection was performed. Zhao et al. (2020) constructed a MN-based glucose biosensor system with a hollow pyramidal MN array template (Figure 4B). The device is composed of three silk/D-sorbitol pyramidal MNs that immobilized platinum (Pt), silver (Ag) wires, and glucose oxidase (GOD) during fabrication. The silk/D-sorbitol complex provided a bio-friendly environment for the enzyme reaction. The mechanical breaking strength could be adjusted by varying the silk to D-sorbitol ratio, thus ensuring that the microneedles can penetrate the skin. The enzymatic amperometric response is linearly related to the glucose concentration. Even at 37°C, the microneedles showed high stability during long-term monitoring and storage.

In addition to solid and hollow MNs, some groups attempt to employ porous MNs for electrochemical sensors. By combining a sponge-like porous PDMS matrix with a hyaluronic acid (HA) coating, the prepared MNs exhibited desirable mechanical properties, and the MNs were able to penetrate the skin and are flexible after insertion into the skin (Takeuchi et al., 2020). It was noteworthy that the reported MN arrays successfully extracted ISF not by capillary action but by repetitive compression. The results demonstrated the applicability of flexible MNs for continuous glucose monitoring.

### Microneedle-Based Surface-Enhanced Raman Scattering Sensor

**FIGURE 5:**
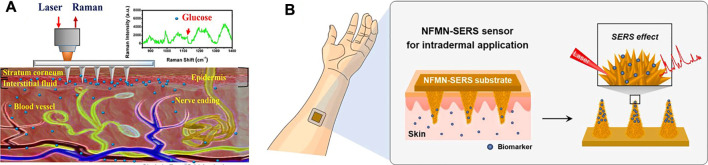
**(A)** Schematic diagram of the transdermal detection of glucose *in vivo* using the F-PMMA MN array based on surface-enhanced Raman spectroscopy. Reproduced with permission (Ju et al., 2020). Copyright 2020, American Chemical Society. **(B)** Scheme of the NFMN-SERS sensor for an intradermal detection. The inset image shows the flower-like nanostructures formed on the MN structure. Reproduced with permission (Li H. et al., 2021). Copyright 2021, Elsevier.

Surface-enhanced Raman scattering (SERS) is noninvasive spectral technology, which is the result of enhanced sensitivity of Raman spectral fingerprint and local surface plasmon resonance (Xu et al., 2019; Shrivastav et al., 2021). The quantification of samples could be realized without sample pretreatments such as dilution or centrifugation, the processing speed is fast, and the measurement is in real-time. The bottleneck problem that SERS directly applied to skin detection is that the penetration depth of the Raman laser is about 200 m under the skin (Krafft et al., 2009), while the depth required for clinically relevant analytics is 700 m. MNs are a good tool to address this issue. Yuen and Liu (2014) used MNs for *in situ* SERS measurements in skin phantoms first. They fabricated Ag-coated MNs as SERS probes for sensitive detection of target molecules at depths beneath 700 μm, simulating the absorption and scattering of light by human skin. Ju et al. (2020) also used silver nanoparticles (Ag NPs) to enhance Raman signals. Following the incorporation of 1-decanethiol to the surface of Ag-coated array, the sensors were calibrated in the 0–20 mM range in a skin model and then tested in a streptozotocin (STZ) mouse model for an *in vivo* quantitative study of glucose in type I diabetes (Figure 5A). The results demonstrated that the functional polymethyl methacrylate (F-PMMA) MN array enabled the direct measurement of glucose in the ISF within minutes and maintained its structural integrity.

In addition to Ag NPs, Au NPs have also been employed for enhancement of the Raman signal in MN-based sensors. Park et al. (2019) reported a SERS probe using MN arrays as a noninvasive sensing platform for ISF. The arrays of MNs were made from a commercial polymer binder and coated by plasmonic Au nanorods modified by a pH-sensitive 4-mercaptobenzoic acid. The sensor could monitor pH values in the range of 5–9 and could detect pH values in agar skin models and in human skin *in situ*. Li H. et al. (2021) prepared a biomimetic NFMN-SERS substrate for sensitive and fast intradermal sensing. Nanoflower structures were fabricated on the basis of hydroxyapatite nanostructures as SERS substrates, and Au nano-islands were synthesized on the petals of nanoflowers, creating highly dense hot spots for the SERS effect (Figure 5B). The petals facing close in the nanoflower structure further generated a plasmonic coupling effect for sensitivity improvement. The developed finite-difference time-domain-SERS sensor is expected to be applied for various intradermal sensing, especially for chemical biomarkers in interstitial fluids of skin.

### Microneedle-Based Colorimetric Sensor

The colorimetric sensors possess advantages including being easy to operate and not needing expensive or complex equipment (Wang et al., 2021b). Especially, the signal changes can be easily monitored for field analysis and bedside diagnosis.

**FIGURE 6:**
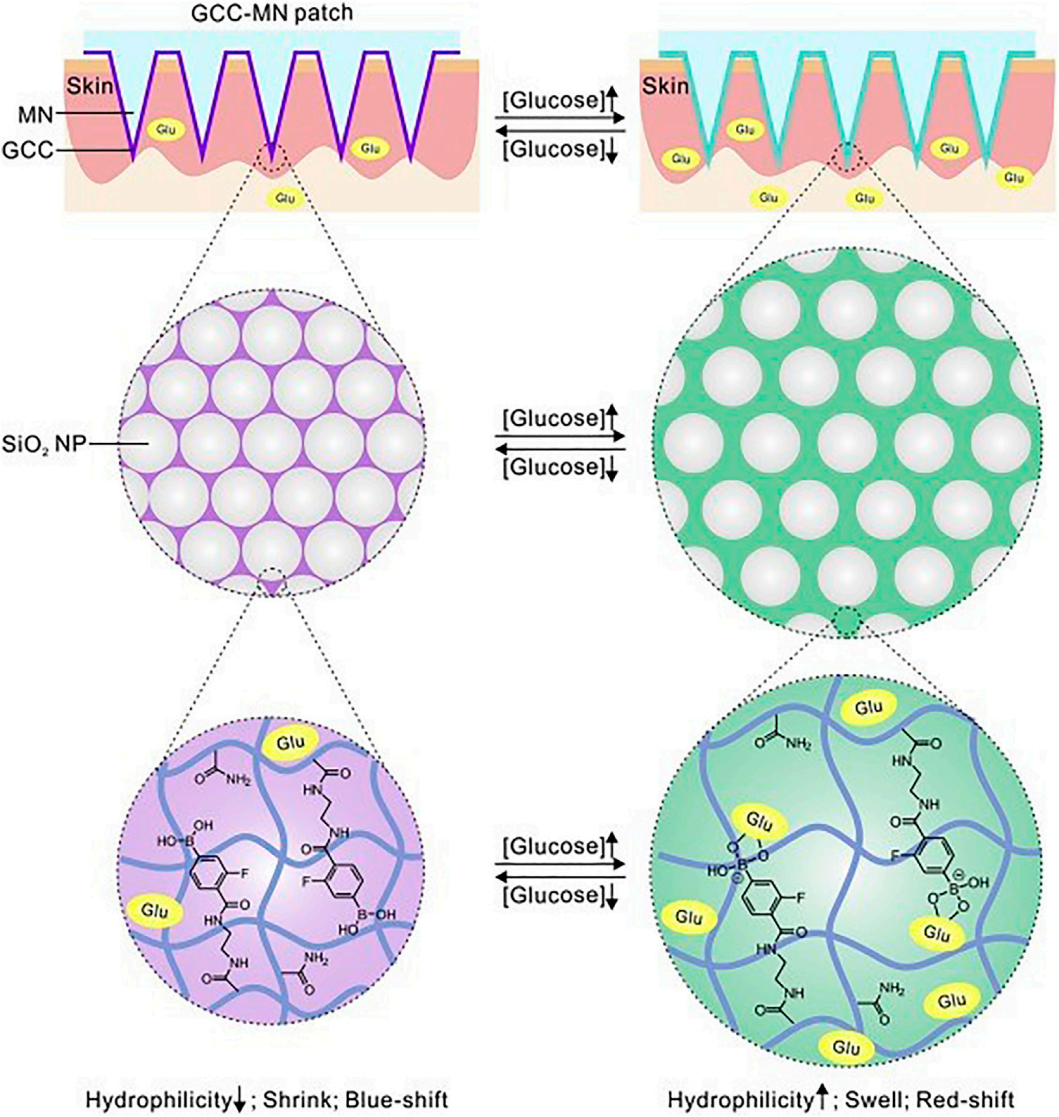
Schematically illustrated the formulation and mechanism of the GCC-MN patch for naked eye monitoring of glucose concentrations. Reproduced with permission (Zeng et al., 2020). Copyright 2020, Elsevier.

Some groups reported a MN-based colorimetric sensor for glucose detection. Nicholas et al. (2018) reported a single hollow MN sensing device that used an enzyme-based colorimetric principle to achieve fast measurement of glucose concentrations in simulated ISF. The glucose sensor was integrated into the paper matrix as a backplane and connected to the hollow MN. The device was capable of rapid extraction of simulated ISF within 5 s and was able to produce a color change related to glucose concentration within 30 s. Zeng et al. (2020) described a minimally invasive colloidal crystal MN patch for macro blood glucose monitoring. Glucose-responsive colloidal crystal (GCC) MNs were constructed on a polymer core that supported the GCC shell for glucose sensing (Figure 6). The GCC MN patch can convert glucose concentration to a reversible color change discernible to the naked eye within 5 min. Being demonstrated in a mouse model of type-1 diabetes, the GCC-MN patch simultaneously realized ISF extraction, glucose sensing, and the resulting glucose-related color changes. An integrated sampling and display of transdermal colorimetric MN patch was also developed (Wang Z. et al., 2020). The color rendering of 3,3',5,5'-tetramethylbenzidine (TMB) was triggered by a cascade of enzymatic reactions of glucose oxidase and horseradish peroxidase (HRP) induced by excessive glucose. The upper layer of HRP was biomineralized with calcium phosphate, which increased pH-responsive properties, improving sensitivity and preventing nonspecific reactions. The colorimetric sensor enables minimally invasive extraction of mouse ISF and rapidly converted glucose levels into visible color changes.

In addition to glucose, MN-based colorimetric sensors have been successfully used for rapidly detecting protein bio-markers in dermal ISF (Jiang and Lillehoj, 2020). Sample collection is facilitated by a hydrophilic hollow MN array that autonomously extracts ISF by surface tension and delivers it to antibody-based lateral flow test strips. The potential enhancement mechanism was elucidated through experimental studies, and a simple gold enhancement treatment was employed to improve the detection sensitivity. For proof-of-concept, the device was used to detect a biomarker of malaria infection, the histidine-rich protein 2 of *P. falciparum*. The device was able to detect a target protein concentration as low as 8 ng/ml. Each test could be completed within 20 min and did not require additional equipment. MN-based colorimetric sensors for detection of multiple health-related biomarkers have also been reported by He et al. (2021). The biosensor displayed color changes in response to changes in biomarker concentrations such as pH, glucose, uric acid, and temperature, which could be read directly with the naked eye or captured by a camera for semi-quantitative measurements. The colorimetric dermal tattoo biosensor has been demonstrated to simultaneously detect multiple biomarkers *in vitro*, *ex vivo*, and *in vivo*, and to monitor changes in the concentration of biomarkers over a long period of time (at least 4 days). Such capacity shows great potential for long-term health monitoring.

## Microneedle-Based Device for Bio-Signal Recording

Both humans and animals produce regular electrical signals in static and active states. Monitoring these biological signals, for example, electromyogram (EMG), electrocardiogram (ECG), and electroencephalogram (EEG), can help to understand human pathology and physiology (Peng et al., 2016). Conventional wet Ag/AgCl electrode could obtain biological signals with relatively high resolution in clinical practice. However, several disadvantages limit its further application. First, Ag/AgCl electrodes always require skin preparation such as haircuts and skin scrapes (Griss et al., 2000). Second, gel electrolytes are often used to reduce the high impedance of the cuticle (Wang et al., 2017). However, the gel may cause skin irritation or allergic reactions that make the subject uncomfortable (Kato et al., 2006). In addition, the gel dries out over time, which reduces the quality of the recorded signal (Tallgren et al., 2005). Therefore, wet electrodes are suggested to fail to long-term bio-signal recording. Compared with wet electrodes, dry electrodes do not require electrolytic gel, and thus can record biological signals continuously (Baek et al., 2008; Fu et al., 2020). However, the electrode-skin interface impedance (EII) is still very high and sensitive to human movement (Yu et al., 2009; Forvi et al., 2012).

**FIGURE 7:**
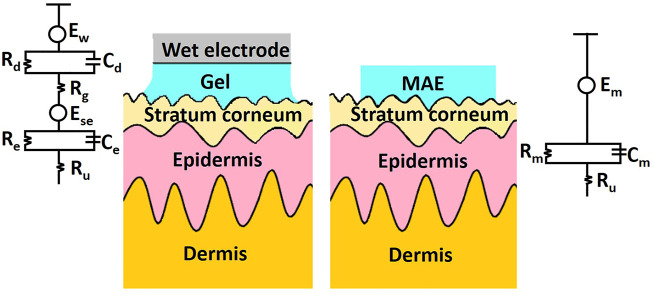
Schematic and electrical equivalent circuit model of wet electrodes and MAE, respectively.

As a kind of dry electrode, the MN array electrode (MAE) was first proposed by Griss et al. (2000). MAE can penetrate the cuticle of human skin and eliminate the effect of cuticle on impedance (Ren et al., 2020b). Meanwhile, MNs do not require skin preparation for puncture and result in minimal skin trauma (Kashaninejad et al., 2021). It has been shown that MAE has a lower and more stable EII than commonly used dry electrodes. Figure 7 schematically showed the electrical equivalent circuit model of wet electrodes and MAE. Here, E_w_ is the half-cell potential. The interface between the wet electrode Ag/AgCl and the electrolytic gel could be simulated by the parallel connection of capacitor C_d_ and resistor R_d_. R_g_ represents the electrical resistance of the gel used in the experiment. E_se_ represents the potential difference between the gel and the stratum corneum. The stratum corneum can also be modeled by a capacitor C_e_ and a resistor R_e_ in parallel. R_u_ represents the resistance of the dermis and subcutaneous tissue. The equivalent circuit of MAE is relatively simple. Since the MNs are in direct contact with the epidermis, there is only coupling between the electrodes and the conductive layers within the skin, except for the conductive resistance of the dermis and underlying tissue, described by the half-cell potential E_m_ and connected in parallel with the resistance R_m_ and the capacitance C_m_.

To date, MN-based devices have been successfully used in ECG, EMG, and EEG recording (Chen et al., 2016; Pei et al., 2017; Ren et al., 2018; Sun et al., 2018; Mahony et al., 2019; Satti et al., 2021; Wang Y. et al., 2021). The properties of MNs for bio-signal recording are summarized in Table 4. Due to the relatively mature processing technology in semiconductor industries, many MN materials are based on silicon. Silicon is the first material used in MN fabrication due to its instinct excellent mechanical strength, good biocompatibility, and the matureness of micro-fabrication technologies (Li et al., 2018). Griss et al. (2000) used silicon MN array for EEG signal recording in 2000. Forvi et al. (2012) also employed silicon MNs that have good performance in recording EEG, EMG and ECG, but silicon electrodes need to be coated with metal to achieve a relatively lower impedance. In addition, the silicon material is hard and lacks flexibility, resulting in the unfitness of electrodes on the body skin, and concomitant motion generated artifact signal (Tachikawa et al., 2010). Wang et al. developed flexible parylene-based MN electrode arrays with silicon MNs, which could provide not only conformal but also robust contact (Wang et al., 2017). Compared with wet electrode, a good impedance density of 7.5 KΩ cm^2^ @10 Hz was attained. Flexibility of P-MNEA contributes to recording stability, and P-MNEA realizes credible EEG acquisition.

**TABLE 4:** Properties of MNs for bio-signal recording.

MN material	MN length/µm	MN shape	MN manufacturing method	Application	Reference
Silicon	300	Pyramidal	Sawing and wet isotropic etching	ECG	Pei et al. (2017)
Silicon	200	Pyramidal	Sawing and wet isotropic etching	EEG	Chen et al. (2013)
Silicon	300	Pyramidal	Wet etching	ECG, EMG, and EEG	Forvi et al. (2012)
316L stainless steel and gold, parylene	550	Square	Etching	ECG, EMG, and EEG	Ren et al. (2020a), Satti et al. (2020), Satti et al. (2021)
Iron nanoparticles and NdFeB microparticles	1,200	Pyramidal	Magnetic field-induced spray self-assembly, thermal curing, and electromagnetization	EMG	Li et al. (2020)
Iron powder and epoxy novolac resin	700	Pyramidal	Magnetization-induced self-assembly	ECG and EMG	Chen et al. (2016)
Iron powder	700	Micro-stalagmite	Magneto-rheological drawing lithography	ECG, EMG and EEG	Ren et al. (2018)
Titanium, SU-8 and gold	500	Small humps at the bottom	Laser machining	ECG and EMG	Sun et al. (2018)
Hard epoxy resin	700	Rectangular pyramid	Mold casting and computer numerical control	ECG and EMG	Hou et al. (2021)
Polyimide	400	Tip	Two-step conformal molding technique, laser cutting, metal sputtering, and electrochemical deposition of the conducting polymer	EEG	Li J. et al. (2021)

**FIGURE 8:**
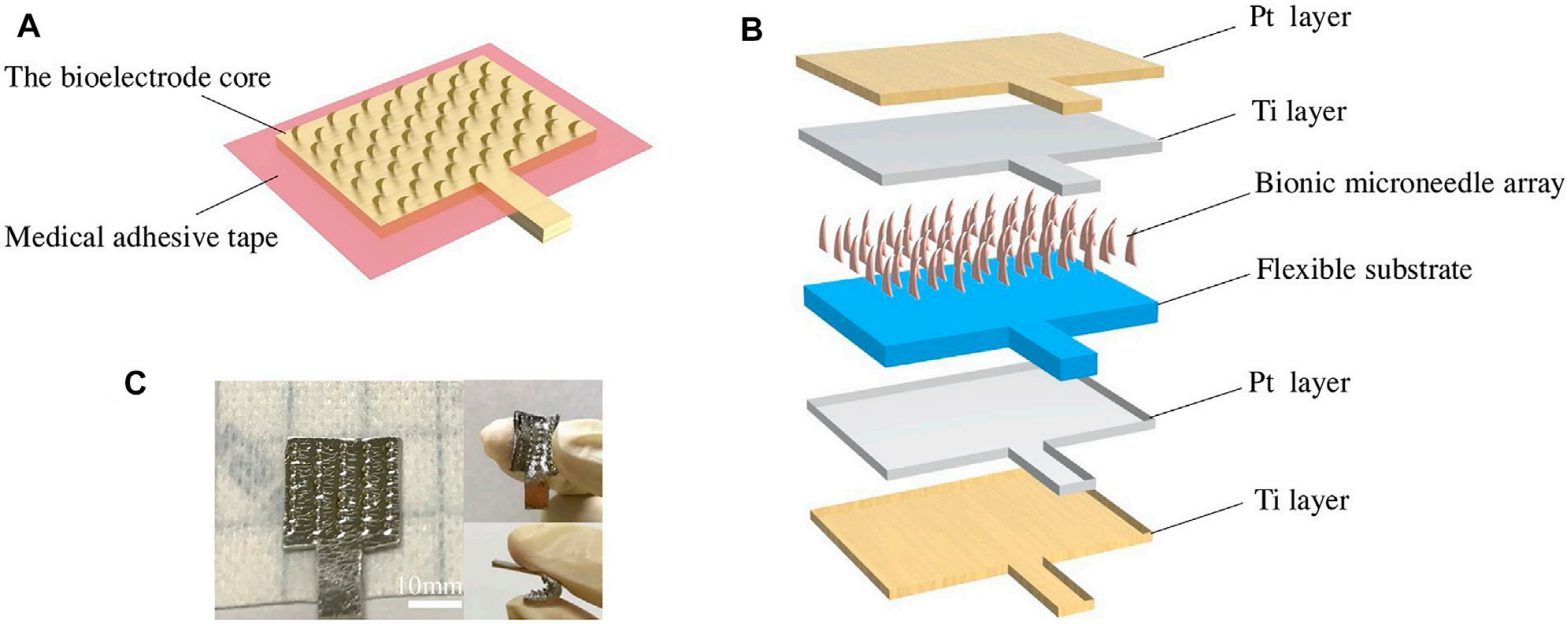
Design of the flexible dry bioelectrode: **(A)** assembling diagram of a flexible dry bioelectrode; **(B)** schematic diagram of an electrode core structure; and **(C)** optical picture of a flexible dry bioelectrode. Reproduced with permission (Li Y. et al., 2021). Copyright 2021, Elsevier.

Metal materials are also good candidate materials for MEA fabrication. For example, MEA with MNs with a length of 550 μm could be constructed with a magnetization-induced self-assembly approach (Wang Y. et al., 2021). The MNs could penetrate the cuticle and reach the human epidermis for high-quality signal recording. In addition, the appropriate MN penetration length will not induce harm to the human dermis; therefore, painless signal acquisition could be achieved. The improved performance of MEA in EMG and ECG recordings has been demonstrated experimentally in both able and amputee subjects. Inspired by the edge of golden margined century plant leaf, a kind of flexible dry biological electrode with MN structure was proposed (Figure 8) (Li Y. et al., 2021). The red copper sheet with 100 µm in thickness was selected as a material for the MN array due to its excellent conductivity and processability. The results showed that compared with the triangular MNs, the curved hook MNs exhibited smaller penetration resistance, larger pullout force, better damage resistance, and stronger bonding force with a flexible substrate. Compared with Ag/AgCl wet electrode, the flexible dry biological electrode with curved hook MN structure showed better time domain and frequency domain performance in EMG signal acquisition.

Apart from silicon and metal, polymers could also be considered for MN electrodes. A novel MN array–based dry electrode modified with a ring-shaped flexible polyimide substrate matrix was fabricated by Li J. et al. (2021), which was compatible with EEG signal recording on hairy scalp. The loop structure allows the hair to be passed through the MN attached to the back. The MN on the “ring” can be pressed from behind to directly touch the scalp. MNs with pointed tips, also based on polyimide, can penetrate the cuticle, the outermost layer of the skin, enabling low-impedance contact and minimal invasiveness. Satti et al. (2020) introduced a rigid parylene-coated MN electrode array for the construction of a portable ECG circuit, which was able to monitor the process of ECG reducing the motion artifacts. In comparison with the traditional Ag/AgCl electrodes, the prepared MNE showed an increased stability and durability for dynamically and long-termed monitoring ECG. The microneedles could survive the compression force until 16 N and successfully penetrate skin tissue with an insertion force as low as 5 N. The electrical properties of the MNE were characterized using impedance spectroscopy under the equivalent circuit model. The resultant wearable wireless MNE demonstrated the potentials for ECG recording with the reduction of the movement artifact noises in the process of dynamic behaviors. A recent case was reported that a flexible MAE with a Miura-ori pattern was constructed for monitoring biosignals accurately and stably (Hou et al., 2021). The mold was directly prepared using high-precision machining. The mold was consisted of micro-level needle tips and a matrix macro-level Miura-ori structure. The flexible M-MAE patches were extruded with a further PDMS mold pressing process. A series of tests of the EII and flexibility supported this minimally invasive M-MAE with outstanding biosignal sensing performance as the result of smaller skin-electrode impedance and more stable sensing signal output against bending. The M-MAE possessed benign airiness due to air-permeable channels, facilitating to get rid of sweat timely and stabilize signal output. The M-MAE sensing patch also showed to have *in situ* practicability and long-term stability to realize real-time biosignal recording of ECG and EMGs.

## Future Challenges and Perspectives

### Challenges of Microneedle-Based Sensors

In the last decades, the advanced properties of MNs have driven the development of MN biomedical applications. However, most of the commercial MNs have focused on delivery of drugs and vaccines (Chen et al., 2016; Lee et al., 2020). Nowadays, a few studies have reported the clinical applications of MNs in continuous glucose monitoring, but the study on MNs for biological analysis is still in the laboratory stage. Thus, the practical biological analysis applications of MNs still require prolonged efforts from both industry and academia. There are several areas for improvement. First, the cost and scale produce of MNs limit their wide industrialization. Second, the invasive sample collection always results in its disqualification for clinical use. The risk of their disease transmission through ISF also exists if they are not properly sterilized. Furthermore, some metals such as nickel may result in severe allergic reactions. Finally, the research on MN-based system mainly consists of *in vivo* detection and *in vitro* detection. Pig skin is usually employed as a model for *in vitro* detection because of its similar histological and biochemical properties to human skin (Swindle et al., 2012). However, pig skin shows a higher permeability than human skin, and inherent interspecific variation and inter-batch variability from skin may result in complicated interpretation of test results (Luo et al., 2016). The *in vivo* model is not ideal due to economic, moral, and legal reasons (Grimm et al., 2019).

### Prospective of Microneedle-Based Sensors

Simpler and more economical micromachining techniques are needed to be developed to achieve a mass production of high-precision MNs. The 3D printing technique holds great potential for scalable MN production. MNs must be made of materials that are harmless and nonirritating. Meanwhile, the MN-based devices must be rigorously disinfected before clinical application to get rid of the microorganisms’ contaminations under the skin surface. Although metal or silicon MNs can usually be easily hyperpyrexia sterilized, polymer MNs can be rendered to deformation under such conditions, and alternative sterilization methods are required. Additionally, due to the complex composition of body fluid, the stability of the MN-based sensors after contact with body fluid should also be assessed. Therefore, further development of materials for the preparation of microneedles is needed in the future. It is necessary to develop easy-to-adjust and versatile *in situ* skin tools for MN assessment. For example, multifunctional animal-free 3D tools have proven their great potential for rapidly and economically evaluating MN devices (Totti et al., 2019). More animal-free, reproducible models will need to be developed in the future for MN-based device verification, and more reliable clinical trials of MNs are highly desirable well in the future. Overall, taking advantage of enabling continuous monitoring in personalized healthcare in a robust, reliable, and noninvasive approach, MNs are a promising candidate to provide an avenue for modern diagnosis through the transdermal route.
